# Supplementary material for: Carbon costs and benefits of Indonesian rainforest conversion to plantations
Source: Nat Commun. 2018 Jun 19;9:2388. doi: 10.1038/s41467-018-04755-y (PMC6008452; doi:10.1038/s41467-018-04755-y)
Supplement: Supplementary file 1 — Supplementary Information [file 41467_2018_4755_MOESM1_ESM.pdf]

Supplementary Information:

“Carbon costs and benefits of Indonesian rainforest conversion to plantations”

Guillaume *et al.*

Supplementary Figure 1: Redundancy analysis constrained by land use (F, rainforest; J, jungle rubber; R, rubber; O, oil palm) and region (B, Bukit Duabelas, H, Harapan). For full names, see Supplementary Table 1.

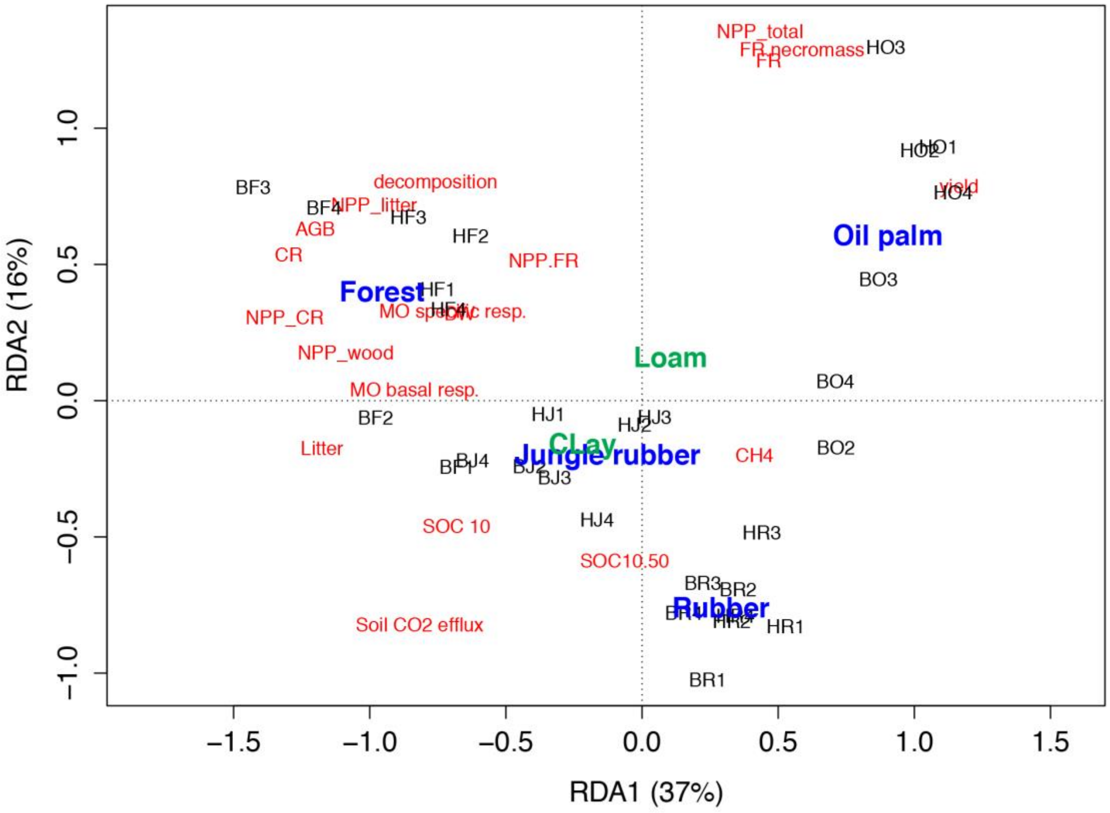

Supplementary Figure 2: Variables contribution to principal components 1 (a), 2 (b) and 3 (c)

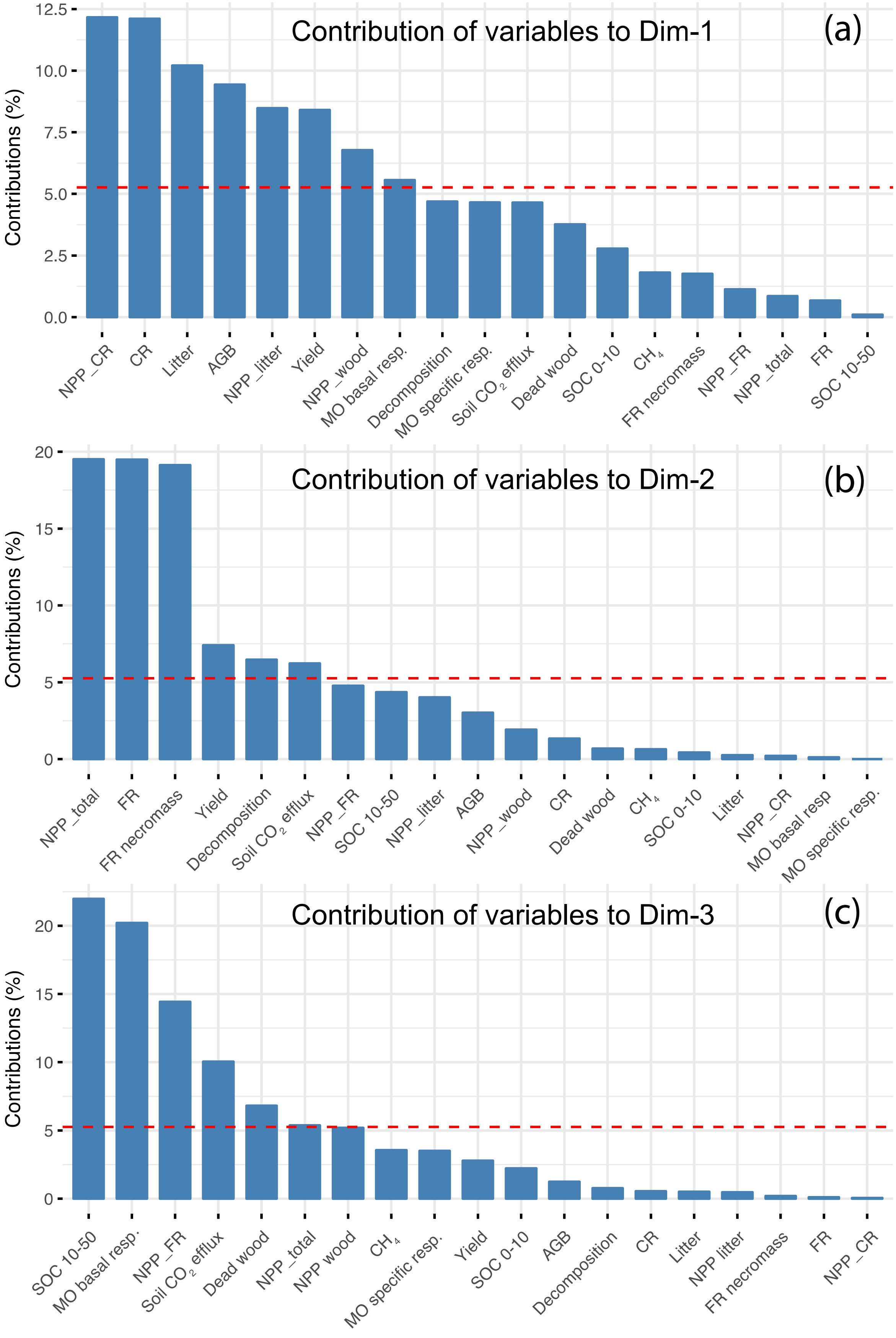

Supplementary Table 1. Mean ± standard error and statistics of carbon stocks, fluxes and decomposition in each land-use.

	Forest		Jungle rubber		Rubber		Oil palm		Df	F-value	P-value	n	Tranformation	Source
**Carbon stocks (Mg C ha^{-1})**														
**Living biomass**														
Aboveground biomass	159.7 ± 11.2	*c*	57.6 ± 2.4	*b*	28.7 ± 3.4	*a*	33.1 ± 2.3	*a*	3,28	93.97	1.0E-14	8	log	Kotowska et al. (2015)*
Coarse roots biomass	32.4 ± 1.4	*c*	16.4 ± 1.0	*b*	8.2 ± 0.8	*a*	5.6 ± 0.4	*a*	3,28	164.8	2.0E-16	8		Kotowska et al. (2015)*
Fine roots biomass	1.4 ± 0.2	*bc*	1.2 ± 0.2	*ab*	0.8 ± 0.1	*a*	2.1 ± 0.3	*c*	3,28	8.7	3.1E-04	8	log	Kotowska et al. (2015)
Total biomass	193.6 ± 12.3	*c*	75.2 ± 3.3	*b*	37.6 ± 4.1	*a*	40.8 ± 2.7	*a*	3,28	105.4	2.3E-15	8	log	Kotowska et al. (2015)*
**Dead Biomass**														
Dead wood	4.6 ± 0.9	*a*	4.4 ± 2.2	*a*	NA		NA			44.00	0.235	8	Wilcoxon (W)	Kotowska et al. (2015)
Litter	7.0 ± 1.1	*c*	4.5 ± 0.3	*bc*	3.1 ± 0.6	*b*	0.8 ± 0.2	*a*	3,28	28.94	1.0E-08	8	log	Krashevska et al. (2015)
Fine roots necromass	1.4 ± 0.3	*a*	1.0 ± 0.1	*a*	0.7 ± 0.1	*a*	2.3 ± 0.2	*b*	3,28	11.26	5.1E-05	8	log	Kotowska et al. (2015)
**SOC**														
0-10 cm	34.9 ± 2.5	*a*	30.1 ± 3.7	*a*	31.4 ± 4.4	*a*	24.0 ± 2.4	*a*	3,28	1.82	0.167	8		Guillaume et al. (2015)
0-50 cm	77.0 ± 4.5	*a*	82.6 ± 7.8	*a*	83.4 ± 10.9	*a*	66.1 ± 6.2	*a*	3,28	1.21	0.326	8	log	Guillaume et al. (2015)
**Total C stocks**	283.5 ± 12.2	*c*	167.9 ± 10.3	*b*	124.9 ± 11.9	*a*	109.9 ± 5.5	*a*	3,28	58.2	3.75E-12	8		
Total C losses	NA		115.6 ± 15.9	*a*	158.6 ± 17.0	*b*	173.5 ± 13.4	*b*	2,21	9.82	9.8E-04	8		
Biomass losses	NA		118.4 ± 12.7	*a*	156.0 ± 13.0	*b*	158.2 ± 12.6	*b*	2,21	33.1	1.3E-07	8		Kotowska et al. (2015)*
Aboveground stocks	171.2 ± 11.6	*c*	66.7 ± 2.7	*b*	31.9 ± 3.0	*a*	33.8 ± 2.3	*a*	3,28	122.5	3.3E-16	8	log	
Belowground stocks	112.3 ± 4.9	*b*	101.2 ± 8.5	*ab*	93.1 ± 10.9	*ab*	76.1 ± 6.1	*a*	3,28	4.51	0.011	8	log	
ratio AG/BG	1.55 ± 0.13	*c*	0.68 ± 0.05	*b*	0.37 ± 0.05	*a*	0.47 ± 0.05	*a*	3,28	38.34	4.8E-10	8	log	
**Carbon fluxes (Mg C ha^{-1} yr^{-1})**														
NPP wood	4.5 ± 0.4	*b*	3.3 ± 0.2	*ab*	2.7 ± 0.3	*a*	3.1 ± 0.3	*a*	3,28	6.85	1.3E-03	8		Kotowska et al. (2015)*
NPP litter	4.5 ± 0.4	*c*	3.7 ± 0.2	*bc*	1.9 ± 0.1	*a*	2.9 ± 0.3	*b*	3,28	27.96	1.4E-08	8		Kotowska et al. (2015)
NPP coarse roots	1.6 ± 0.1	*b*	1.2 ± 0.1	*ab*	0.5 ± 0.1	*ab*	0.3 ± 0.0	*a*	3,28	26.08	9.2E-06	8	Kruskal-Wallis (Chi^2)	Kotowska et al. (2015)*
NPP fine roots	1.1 ± 0.1	*a*	0.9 ± 0.1	*a*	0.7 ± 0.1	*a*	0.8 ± 0.2	*a*	3,28	1.53	0.229	8		Kotowska et al. (2015)
NPP yield	NA		0.5 ± 0.1	*a*	2.0 ± 0.5	*a*	10.0 ± 0.8	*b*	2,21	18.74	8.5E-05	8	Kruskal-Wallis (Chi^2)	Kotowska et al. (2015)
NPP eco	11.7± 0.6	*c*	9.2 ± 0.5	*b*	5.8 ± 0.3	*a*	7.1 ± 0.5	*a*	3,28	28.59	1.1E-08	8		
**NPP total**	11.7 ± 0.6	*b*	9.6 ± 0.5	*ab*	7.8 ± 0.4	*a*	17.1 ± 0.9	*c*	3,28	42.05	1.7E-10	8		Kotowska et al. (2015)*
CO_2 emission	16.6 ± 0.8	*b*	15.8 ± 0.6	*b*	16.3 ± 0.9	*b*	9.8 ± 0.5	*a*	3,28	21.01	2.5E-07	8		Hassler et al. (2015)
CH_4 uptake (*1000)	-1.9 ± 1.1	*ab*	-2.1 ± 0.3	*a*	-0.6 ± 0.2	*b*	-1.0 ± 0.3	*ab*	3,28	8.32	0.0398	8	Kruskal-Wallis (Chi^2)	Hassler et al. (2015)
**NPP allocation (% of NPPenv)**														
NPP wood	38 ± 2	*a*	37 ± 2	*a*	45 ± 3	*a*	43 ± 3	*a*	3,28	2.94	0.0503	8		
NPP litter	39 ± 2	*a*	41 ± 2	*a*	35 ± 3	*a*	41 ± 2	*a*	3,28	1.79	0.171	8		
NPP coarse roots	14 ± 1	*c*	13 ± 1	*c*	8 ± 1	*b*	5 ± 0	*a*	3,28	54.33	8.5E-12	8	log	
NPP fine roots	9 ± 1	*a*	10 ± 1	*a*	12 ± 1	*a*	12 ± 3	*a*	3,28	3.73	0.292	8	Kruskal-Wallis (Chi^2)	
**C decomposition**														
Litter decomposition (%)	89 ± 1	*b*	58 ± 5	*a*	51 ± 5	*a*	60 ± 11	*a*	3,27	14.33	2.5E-03	8	Kruskal-Wallis (Chi^2)	Krashevska et al. (2018)
Microbial respiration (µg O_2 g^{-1} soil h^{-1})	6.3 ± 0.5	*b*	5.9 ± 0.6	*b*	4.1 ± 0.4	*a*	4.5 ± 0.7	*a*	3,27	7.34	0.0009	8		Krashevska et al. (2015)
Microbial specific respiration (µl O_2 mg^{-1} C_{mic} h^{-1})	14.0 ± 1.2	*b*	11.8 ± 1.1	*ab*	10.7 ± 1.1	*a*	11.1 ± 1.5	*a*	3,27	3.67	0.0239	8		Krashevska et al. (2015) Clough et al. (2016)

* data updated using allometric equations from Chavez et al. (2014) for forest, jungle rubber and rubber, and using allometric equations form Khasanah et al. (2015) for oil palm

Supplementary Table 2. Contribution of soil heterotrophic (Rh) and autotrophic (Ra) CO_2 in total soil CO_2 efflux (Rs) at which ecosystems or SOC are at equilibrium.

	ecosystem equilibrium			SOC equilibrium: maximum fRh			
**Landuse**	**fRh**[a]	**Ra**[b]	**Rh**	**fRh**	**Ra**	**Rh**	**fNPPt**[c]
Forest	0.44 ± 0.03	9.4 ± 0.9	7.2 ± 0.5	-	-	-	-
Rubber	0.36 ± 0.03	10.6 ± 1.0	5.8 ± 0.3	0.19 ± 0.01	13.2 ± 0.9	3.1 ± 0.1	0.55 ± 0.03
Oil palm[d]	0.45 ± 0.04	5.5 ± 0.7	4.2 ± 0.3	0.12 ± 0.02	8.6 ± 0.5	1.1 ± 0.2	0.16 ± 0.03

[a] ratio Rh/Rs
[b] Mg C ha^{-1} y^{-1}
[c] Maximum fraction of NPP resulting in soil C input = (NPP eco– NPP wood) / NPP eco
[d] In oil palm, SOC equilibrium is applicable to the whole surface area except frond pile, soil C input = (NPPfr+NPPcr) / NPP eco.
